# Person-centred leadership in residential care for older people as described by first-line managers

**DOI:** 10.1186/s12912-025-03354-9

**Published:** 2025-06-23

**Authors:** Marie Jönsson, Anna-Karin Edberg, Anneli Orrung Wallin, Malin Sundström, Annica Backman

**Affiliations:** 1 Research Platform for Collaboration for Health, Faculty of Health Science, Kristianstad University, Kristianstad, SE 291 88 Sweden; 2 Department of Nursing, Umeå University, Umeå, Sweden

**Keywords:** First-line manager, Leadership, Person-centred

## Abstract

**Aims and objectives:**

To explore person-centred leadership in residential care from the perspective of first-line managers.

**Background:**

Studies have shown that person-centred leadership is important for leading person-centred care. Although person-centred care is the current model of care, there is limited knowledge of what person-centred leadership entails in the context of residential care for older people.

**Methods:**

This was an exploratory qualitative study using focus groups and individual interviews with 21 first-line managers in residential care facilities for older persons in five municipalities in Sweden. Data were collected from October 2021 to March 2022. Data were analysed using conventional content analysis.

**Findings:**

Person-centred leadership entailed being person-centred as a leader, focusing on the relationship with personnel, and leading person-centred care by highlighting the older persons’ needs.

**Conclusions:**

Being person-centred as a leader involves acknowledging the personhood of personnel in words and actions, which has the potential to promote person-centred practices. Person-centred leadership entails promoting personnel to meet older person’s changing needs, which must drive operations, while simultaneously trying to meet the needs of personnel.

**Supplementary Information:**

The online version contains supplementary material available at 10.1186/s12912-025-03354-9.

## Introduction

Several challenges are encountered in the care of older people today, both in Sweden and internationally, such as lack of trained personnel, high personnel turnover, staffing shortage, large numbers of personnel managed by first-line managers, and caring for older people with increasingly complex needs in residential care facilities (RCFs) [1, 2]. These challenges place high demands on first-line managers in RCFs; in addition to these organisational and structural challenges, first-line managers are also expected to lead person-centred care [3]. Leading person-centred care entails having one’s own understanding of the person-centred concept and the ability to transform it into practical work in RCFs [4]. A study in a hospital reported that person-centred leadership can be defined as leadership working towards a shared vision or common goal with the involved health-care personnel [5]. We have, however, sparse knowledge of what person-centred leadership entails in the context of residential care for older people, knowledge that is crucial as a basis for developing the concept.

## Background

Person-centred care has become increasingly established and is now prevalent in health care [6]. Person-centred care has had great impact, and relevant steering documents and policies have been developed internationally [3] and in Sweden [7]. In a cluster-randomised controlled trial including older people with dementia, person-centred care improved the quality of life of those cared for [8]. In a cross-sectional study, residents receiving higher levels of person-centred care were rated as having higher quality of life and better ability to perform activities on a daily basis than residents of units with lower levels of person-centred care [9]. Other studies have shown that person-centred care was associated with higher levels of work satisfaction and better teamwork among direct-care staff [10].

There are several conceptualisations of person-centred care. One of the most common is McCormack and McCance’s [11, 12] model, described as a framework in which organisation and leadership are stated prerequisites for conducting person-centred care. Ekman et al. [13] developed a model in the Swedish context that emphasises partnership, patient narratives, and documentation, while Brooker [14] developed a practical model primarily designed to function in the context of people with dementia. The model is based on four key elements: [1] the absolute **v**alue of human lives; [2] an **i**ndividualised approach; [3] understanding the world from the perspective of the service user; and [4] providing a social environment that supports psychological needs (VIPS) [14]. All these models of person-centred care describe the relationship between caregivers and those cared for as central, but McCormack and McCance [12] and Brooker [14] focused more on organisational aspects and conditions emphasising the importance of leadership. These different conceptualisations share the same humanistic values, stressing the importance of seeing the person behind the illness [11, 13, 14]. However, none of the models specifically emphasises leadership or presents a practical approach to leading person-centred care, nor do they offer a clear definition of person-centred leadership.

The field of research on leadership is extensive, and several typologies and theories have been developed in management science or in the context of industry. As Alvesson and Kärreman [15] pointed out, context is regularly neglected. This implies that theories and models cannot easily be transferred to other settings, such as residential care for older people. This is in line with the conclusions of a narrative review by Zonneveld et al. [16] emphasising the need for a stronger focus on leadership behaviours related to the specific context rather than different leadership styles. Bondas [17] also highlighted the problem of transferring, to the health-care context, models of leadership not developed in connection with the patient and the meaning of health care. Research on person-centred care has shown that leadership and person-centred care are associated, suggesting that leadership is a prerequisite for person-centred practices [18–20]. However, in terms of empirical studies, a narrative review synthesising leadership styles in RCFs showed that the relationship between leadership and person-centred care was described in only three of 44 publications [16]. It was also noted that more recent publications have moved away from leadership styles to focus more on leadership behaviours essential for developing RCFs [16]. A study based on personnel-reported data showed that managerial leadership characterised by experimenting with new ideas, monitoring work closely, relying on subordinates, coaching, giving direct feedback, and handling conflicts constructively was associated with person-centred care and psychosocial climate [18]. At the five-year follow-up, leadership was still significantly associated with person-centred care, and the association between leadership and person-centred psychosocial climate was even stronger than initially found [21]. Another study examining the implementation of person-centred care in RCFs described how leadership, from the perspective of personnel and first-line managers, should have an active role and a clear vision taking account of all personnel [22]. A study based on personnel-reported data showed that highly person-centred units in RCFs characteristically had first-line managers who supported personnel provide care based on the individual older person’s needs, engaged with the knowledge of personnel, and fostered professional development [23]. It was also shown that first-line managers were more likely to improve the team spirit and were aware of the care quality provided by personnel in highly person-centred units, suggesting that first-line manager engagement and support are essential for person-centred practices [23]. Previous research reveals that different types of leadership have been associated with person-centred care. From the perspective of personnel, it is important that first-line managers should be committed, supportive, and interested in the development of personnel. From the perspective of managers, an interview study showed that leading person-centred care entails establishing trust and responsibility by delegation, clarifying team roles and positions, taking the personal and relational competencies of staff into account, and creating forums and structures to support person-centred care [4]. This suggests that leadership is crucial when promoting person-centred care, although further knowledge is needed.

In summary, the literature highlights the importance of leadership for person-centred care but does so mostly from the perspective of personnel. Little is known about the perspective of first-line managers, even though person-centred care is the prevalent model and philosophy of care, and it is their responsibility to implement it. Our knowledge of the meaning of person-centred leadership from the perspective of first-line managers is limited and needs to be recognised and conceptualised when developing person-centred practices. This study attempts to address this knowledge gap.

### Aim

This study aims to explore person-centred leadership in residential care for older people from the perspective of first-line managers.

## Materials and methods

### Design

This study is part of a larger project, Person-Centred Care and Leadership in Residential Care (PERLE), exploring person-centred care and leadership among RCF leaders in Sweden. The study has an exploratory qualitative design based on digital interview data analysed inductively. We followed the Consolidated Criteria for Reporting Qualitative Research (COREQ) [24] in presenting this study.

### Setting

In Sweden, RCFs are a special type of accommodation used when care needs are great and personal support is required around the clock. Most older people living in RCFs have multiple comorbidities and many also have cognitive impairment and therefore need specially adapted care and attention. In 2021, about 29% of people aged 80 years or above resided in RCFs [25]. In Sweden, 79% of RCFs are publicly provided and approximately 21% are privately provided, but all are covered by the same legislation and are tax financed [26].

The professions of RCF first-line managers vary, and among the approximately 9000 RCF managers in Sweden, 86% of whom are women, the most common professions are social worker (41%) followed by registered nurse (25%) [27]. First-line managers have operational, financial, and personnel responsibilities [28]. They also have responsibility for quality of care and value-based work [27]. Registered nurses in the same context are responsible for leading nursing care [25, 29]. On average, a first-line manager of an RCF in Sweden is responsible for approximately 60 personnel [30]. Responsibilities of RCFs are mainly regulated by two laws in Sweden: the Social Services Act [31] and the Health and Medical Service Act [32]. The Social Services Act states that the municipalities are responsible for RCFs, which are a means-tested form of housing. The Health and Medical Service Act, on the other hand, covers health-care activities and involves registered personnel.

### Sample

The sample comprised 21 first-line managers from five municipalities. The sample was purposively selected to include people who could report relevant experiences and describe the phenomenon being investigated [33]. With permission from the heads of social services in five municipalities, first-line managers were invited to participate in the study. They were informed about the study at digital workplace meetings. The first author also presented the research topic and the motivation for the study at these meetings, during which time written information was also given out. The municipalities varied in size, and both rural and urban areas in southern and northern Sweden were represented. The inclusion criterion was: being employed as a first-line manager at an RCF for older people in one of the included municipalities. Interested first-line managers contacted a member of the research team. In total, 24 managers agreed to participate in the study, but three dropped out due to a heavy workload. All participants were invited to focus-group interviews, but due to pandemic-related and logistical issues, the participants were also offered individual interviews. The final sample comprised 21 first-line managers who participated in focus groups (*n =* 7) with two to four participants each, or in individual interviews (*n =* 3). Twenty of the participating first-line managers worked in public RCFs, and one worked in a facility run by a private provider. The focus groups comprised first-line managers of different RCFs, but commonly from the same municipality. The participants had a mean age of 44 years, and 19 were women and two were men. For a detailed description of the sample, see Table 1.

**Table 1:** Characteristics of participants

Characteristics	*n* = 21
Age, yrs	
Range (md)	24–62 (49)
Gender	
Women/men	19/2
Profession^1^	
Social worker	9
Registered nurse	7
Other education	4
Work experience as first-line manager^1^	
Range, yrs (md)	1–40 (7.5)
Leadership education^1^	
Yes/no	15/5
Education in person-centred care^1^	
Yes/no	8/12
Type of care facility^2^	
General residential care facility	9
Dementia care facility	10
Short-term accommodation	3
Other	2
Number of residents at the facility^1^	
Range (md)	16–102 (30)
Number of personnel at the residential care facility^1^	
Range (md)	12–120 (35)

^1^ Missing data, *n* = 1, ^2^ More than one alternative possible

### Data collection

Prior to data collection, two pilot focus-group interviews were conducted. The first pilot interview focused on testing the interview guide while the second focused on the digital technology. The pilot interviews were not included in the study as the participants worked in a different setting. Data were collected from October 2021 to March 2022. Prior to the interview, first-line managers were asked to provide demographic information in digital form. The same interview guide, with open-ended questions, was used in all interviews. The first author moderated all focus-group interviews and the fourth or fifth author was the assistant moderator. All interviews were conducted digitally via Zoom or Teams with the participants located at their workplace or home. The moderator facilitated the discussion and ensured that all participants were involved, whereas the assistant moderator took notes, posed complementary questions, and summarised the discussion so that the participants could reflect on it [34].

The first author performed the individual interviews. As an introduction, at the start of all interviews the first author explained the purpose of the study and the participants were asked to describe their professional role. All interviews started with an initial question asking participants to elaborate on their experience of person-centred care, to lead the participants towards the interview topic of person-centred leadership. This first part of the interviews on person-centred care are to be published elsewhere. For this study’s purpose the following questions were asked: “Can you tell me about what you think of when you hear ‘person-centred leadership’?” and “What is your experience of leading person-centred care?”. Probing questions were asked, for example: “Can you tell me about a relevant situation?”; “Could you elaborate?”; and “Could you tell me more?”.

The focus-group interviews lasted 115–147 min while the individual interviews lasted 69–73 min. The recorded interviews were transcribed verbatim and anonymised. The moderator and assistant moderator had no relationship with the participants prior to the study.

### Data analysis

Data were analysed using conventional qualitative content analysis as described by Hsieh and Shannon [35]. All authors read the transcribed interviews individually and reflected on their first impression of the text. All authors are women and registered nurses. The first author is a PhD student, while the four co-authors have PhDs in nursing and conduct research in the field of care for older people. The author group has extensive experience in conducting qualitative research. The first author then read the transcripts several times, highlighting text describing person-centred leadership using NVivo software (version 1.6.1) [36]. Line-by-line coding with a focus on meaning units based on person-centred leadership and participants’ experiences of leading daily work was conducted as the initial phase of the content analysis by the first author. When all the data were coded, the codes were grouped into 14 meaningful clusters. These clusters were then sorted by content and grouped into subcategories by the first and fifth authors. The subcategories with similar content were then abstracted into two main categories. For illustrative examples of the analytical process, see Table 2. During the analysis, there was constant iteration between the text, codes, clusters, and possible categories. All authors were involved in this last phase of the analysis.

**Table 2:** Examples of the analysis process based on Hsieh and Shannon (2005)

Transcribed text	Code	Meaningful cluster	Subcategory	Category
*“It’s so important to see the personnel*,* feel needed*,* and try to adjust their work – if you like being with the older persons and having coffee*,* your colleague can do the laundry.”*	To see the personnel and feel needed	To see the underlying human and know their background	Being genuinely interested and showing consideration to personnel as persons	Being person-centred as a leader by focusing on the relationship with the personnel
*“I think it is important that you stand for what you do and that you are clear and that you live as you learn– that is*,* that you are a role model for the personnel in how they*,* for example*,* treat the older persons by always greeting the older person first.”*	Important that you live as you learn and are a role model	To be a role model for the personnel in how you meet the older person	Being a role model	Leading person-centred care by highlighting the older persons’ needs

### Ethical considerations

All procedures performed in this study were guided by the Declaration of Helsinki [37]. The participants reported their interest directly to the researchers, reducing the risk of pressure from colleagues or other managers. It was emphasised to the participants that participation in the study was voluntary, and that they could withdraw from participation at any stage without stating a reason. There was a risk that the study might have intruded on the participants’ working time, which would otherwise have been used to support the older persons and the personnel. However, this was balanced by the fact that the managers’ participation in the study would contribute new knowledge that could clarify their role as first-line managers. The decision to complement the focus-group interviews with individual interviews due to the pandemic situation was a way to allow all first-line managers interested in participation to be heard.

After the interviews, a reflection round was conducted to capture potential questions and concerns in connection with the participants’ discussion of their work, and all participants were invited to contact the first author if any relevant thoughts or concerns emerged after the interview.

### Rigor and trustworthiness

In a qualitative study, trustworthiness is based on credibility, transferability, dependability, and confirmability [38]. To strengthen credibility, the sampling strategy and data collection method were designed to address the aim of the study, recruiting first-line managers to explore person-centred leadership from their perspective. Participants’ ages, experience of management, and educational qualifications varied widely, and the rich interview data revealed a range of experience in various settings across the country. Member-checks were conducted with non-participating first-line managers in participating municipalities by sharing and discussing the findings. The same interview guide was used in all interviews, both focus group and individual, and to enhance dependability, the analysis phase involved senior researchers with experience in content analysis who were not involved in the interviews. The study design and data collection procedure used in this study has been described as strengthening dependability. Quotations from the interviews have been cited to increase the confirmability of our findings and to ensure that the results are based on the participants’ experiences and not the researchers’ preconceptions. Concerning the transferability of the study’s findings, the study was performed in the Swedish context in five municipalities, in order to obtain variation in participants’ experiences of the investigated topic. It is important to consider that the organisation of special accommodations can vary between contexts and countries, which may suggest that the findings could be applied across similar contexts. When transferring the results to other settings, it is thus important to take contextual aspects into consideration.

## Findings

**Fig. 1:**
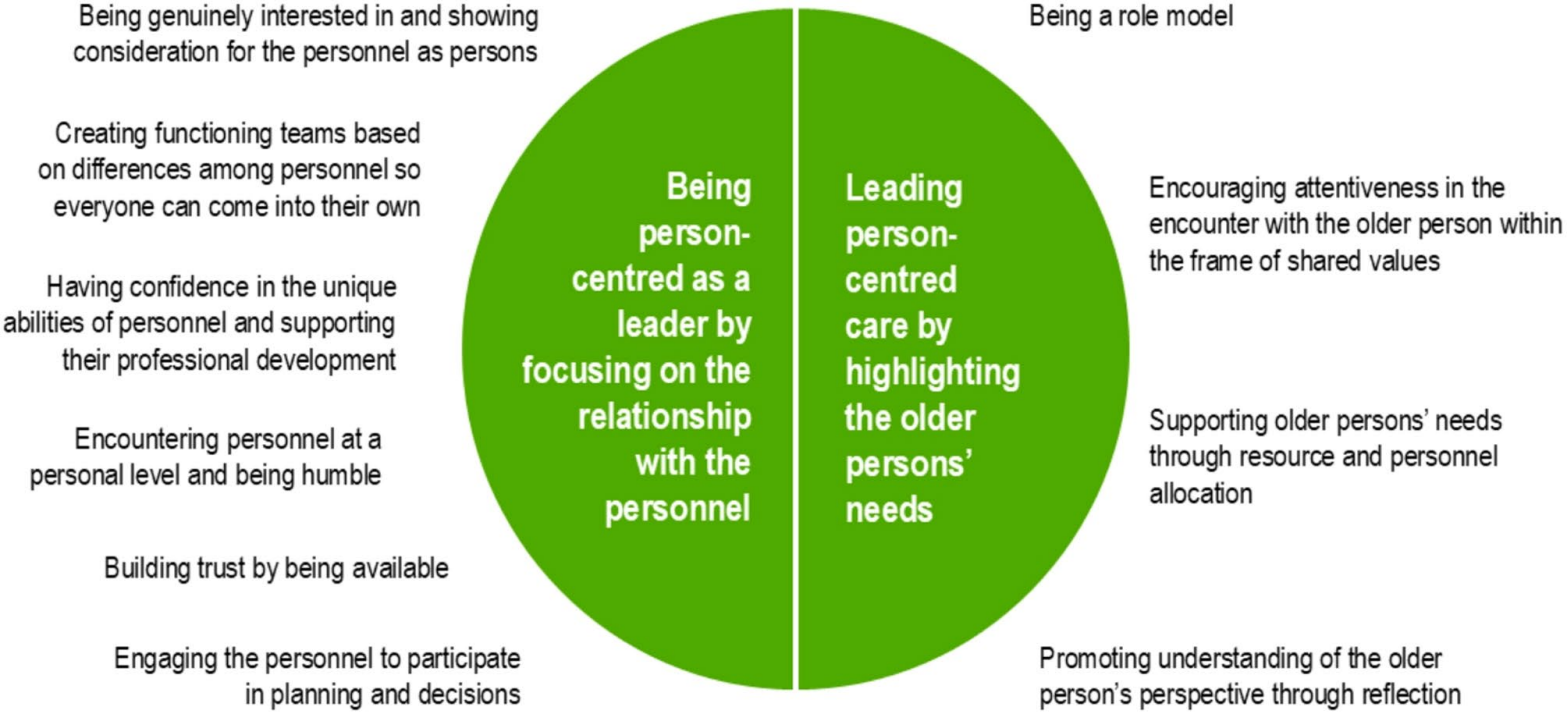
Person-centred leadership from the first-line managers’ perspective

To accommodate variation, person-centred leadership in RCFs for older people from the first-line managers’ perspective can be understood in two main categories and 10 subcategories (see Fig. 1). Fictive names are used in the quotations.

Person-centred leadership consists of two parts that together form a whole: being person-centred as a leader by focusing on the relationship with the personnel; and, simultaneously, leading person-centred care by highlighting the older persons’ needs. This reveals the complexity of person-centred leadership, which can be seen in relation to first-line managers’ two-fold responsibility for personnel, requiring a relationship with them, and simultaneously for the care and its quality, with the older persons and their changing needs in focus.

### Being person-centred as a leader by focusing on the relationship with the personnel

According to the first-line managers, relationships with personnel were created by being genuinely interested in and supporting their professional development. Encountering personnel on a personal level and being available by being physically present at the unit were important for creating such relationships. Being person-centred as a leader also involved engaging the personnel in planning and decision-making, to convey that these were joint processes. It was considered important that the person-centred approach should emanate from within the first-line management, and that this should be conveyed to personnel.

#### Being genuinely interested in and showing consideration for the personnel as persons

The first-line managers highlighted the importance of seeing the person in each of the personnel, of being genuinely interested in and curious about the person behind the professional role, and of adapting and changing the leadership based on personnel needs, although it was sometimes difficult to find time for this. They described how they strove to be sensitive and see the personnel for who they were, without judging or categorising, but instead learning about their backgrounds. To achieve this, they explained how they would ask questions, be attentive, and, importantly, acknowledge the personnel both in conversations with them (e.g., during staff evaluation) and in daily conversations at the unit. Other examples given were showing interest in staff members’ lives and family situations, for example, remembering that they had their children every two weeks or which days their children had hockey practice. It was also described as important to call personnel in case of sick leave, to offer help and express that they were important and missed:

It’s so important that the staff should be seen and feel needed, otherwise it’s as if I can skip coming to work [because] I don’t serve a purpose and am not important. (Rio, FG 7)

I agree with you there…so that…in a way it’s good to talk to the staff and make them feel needed and…make them feel that we mean it. (Sasha, FG 7)

#### Creating functioning teams based on differences among personnel so everyone can come into their own

The first-line managers described how they strove to adapt their leadership style to create functional teams with a strong sense of community by considering how different individuals would best fit in the group. They tried to design the groups through blending different personalities, combining creative, structured, and rigid personnel. Sometimes changes in the group composition were required so that everyone could come into their own or if the group was not functioning well. They also explained how they tried to see staff members’ strengths and weaknesses, and tasks were assigned so that staff could come into their own. One example given was when a staff member who grew up in extreme poverty, and therefore valued cleanliness, wanted to work in the laundry room. That staff member considered the cleanliness of the older people to be paramount, so they were given that position if it was possible. On the other hand, other personnel might prefer relationship-building, so having coffee with the older people suited them better. Other examples were letting personnel who enjoyed baking with the older person bake, while personnel who liked reading the newspaper with the older person could do that instead:

To create a sense of team spirit and for the team to work, it is necessary for everyone to be validated, and that means seeing the person, just as my colleagues Dani and Michele said. Who are they? What are they good at? Everyone is different and everyone is good at different things, but that is what also makes the team function at its best. (Alex, FG 2)

#### Having confidence in the unique abilities of personnel and supporting their professional development

As a person-centred leader, it was considered important to have confidence in the development potential of personnel. According to the first-line managers, it was important to help personnel develop their professional role and try to demonstrate to them that they were not alone in their work. This could be done by explaining to the personnel that they were not interchangeable when mistakes were made, or when they lacked the necessary knowledge for a task in caring for the older people:

As Rio also mentioned before, it is important to make them feel that they are needed and that they are not interchangeable. (Sasha, FG 7)

And I just– well it’s not about that, I don’t want to replace you and bring in someone else from another unit, but I want you to improve. You have these things that you need to work on, and I’m here to help you.… So now we can work together on it! And it’s important that one doesn’t feel alone in it! (Rio, FG 7)

Another way to show confidence in personnel was not to interfere in their day-to-day work but instead to offer support only if needed. They further said that support should be based on the staff members’ unique abilities and needs, for example, offering step-by-step instructions on how to respond to older people with dementia, or offering personnel with language difficulties the opportunity to participate in digital language training to improve their language skills. They described the importance of striving to encourage personnel with advanced knowledge of dementia or technology to use it and share it with other colleagues when possible. Another way to be person-centred as leader, as explained by first-line managers, was to support personnel in their professional development by offering uneducated personnel study time during working hours, if they had the financial means, to achieve a nurse assistant qualification.

#### Encountering personnel at a personal level and being humble

Being person-centred as a leader meant encountering personnel at a personal level despite their different assignments and professional roles. The first-line managers highlighted that qualities such as being both humble and confident were important in contacts with both personnel and older people’s relatives. For example, they needed to be humble given that the personnel had more first-hand knowledge of the older people than they did. The first-line managers expressed how they also wanted to demonstrate that they were human, just like their personnel, and that they too could make mistakes. Humour and joking could also be a way to encounter staff on a personal level and meet them as humans, as long as it did not become too frivolous. The participants also said that they wanted openness from their personnel, and that they appreciated feedback if they had done something wrong to avoid misunderstandings:

Yes, I was really the worst boss, I was so unclear, I just babbled around.… So, I said, I’m really glad, thank you so much for coming and telling me. (Cleo, I 3)

This was especially important when the first-line managers supervised personnel from other cultures, to show that they were all “in the same boat” and there for the same reason, but with different assignments. Cultural differences could sometimes result in unequal relationships, such as subservience to and respect for superiors.

#### Building trust by being available

The first-line managers described how being person-centred as a leader was conveyed by building trust and being available as a leader, for example, by sitting with one’s office door open to show availability. Most importantly, according to the first-line managers, was being present in the units. They described how they strove to find time to visit the units several times a day, sometimes regularly after staff coffee breaks and sometimes spontaneously when the opportunity arose, to enable discussion of situations, to listen, and to see what was going on. Being available also promoted trusting relationships by enabling ordinary conversations, for example, about what the personnel did at the weekend, as well as formal meetings discussing specific situations that had arisen. Working from home as a first-line manager during the Covid-19 pandemic was described as worsening relationships with personnel. Not all of them followed the work-from-home recommendation, as they wanted to show their personnel that they were available in order to build trust. When trust and good relationships were established, the managers said that they could provide functional leadership without being physically present:

You also have to build relationships and be on the unit, because it’s connected a little bit. You can’t just sit by yourself and think that you’re building a relationship. (Eli, FG 5)

Hm, yes, I agree with you, Eli, what you’re saying. And then I also think it is important to be visible out there and, like, take time… for these conversations. (Kim, FG 5)

#### Engaging the personnel to participate in planning and decisions

Being person-centred as a leader, as described by the first-line managers, included inviting personnel to participate in planning and decisions, which made changes easier to implement as they were based on joint decisions. For example, the managers said that involving the personnel in the summer planning and when hiring temporary workers meant that if these processes did not turn out well, at least they were based on joint decisions:

I have hired temporary staff and they have sort of… they have been able to plan the schedule together, the summer schedule… It has gone better and worse some summers, anyway, but then one way or another it screws up, as it does sometimes for one or another reason, then it is still on us that we have failed in some way. (Tory, FG 4)

The first-line managers also gave examples of how they involved staff in workplace meetings. They did this not just to break up managers’ monologues, but also, by trying to draw out quieter personnel, to ensure that assertive individuals did not dominate the discussion. However, not all personnel felt comfortable participating and coming up with their own ideas for developing person-centred care, so it was considered equally important to respect that reticence. They pointed out how they strove to make the personnel feel that it was their workplace, and that joint decisions were intended to make the personnel feel that the work had been planned jointly.

### Leading person-centred care by highlighting the older persons’ needs

Leading person-centred care by highlighting the older persons’ needs involved being a role model for a person-centred approach in encounters with and caring for the older people. The first-line managers described how this involved supporting the older person’s changing needs through responsive resource allocation, i.e., as the older person’s needs changed, resources were reallocated, encouraging responsiveness within the frame of shared values. Furthermore, first-line managers described using joint reflection with personnel to improve their understanding of how their actions could affect the older person.

#### Being a role model

In leading person-centred care, the first-line managers gave examples of how they strove to be role models for the personnel by, for example, always greeting the older people first, and then the personnel, when entering the unit. They said that it was important to be consistent in word and deed. For example, they could help an older person to take a shower, accompany them on a walk, and have a conversation with them to set an example:

Then I change and go and… yes… [help them] take a shower and serve breakfast and… well, take out clothes and help with toilet visits and everything and that… we are actually here for one reason only, to help the residents. (Valentin, FG 1)

It’s so good, Valentin, but I also agree with you, so it’s really important that you are out in the care unit. (Nino, FG 1)

Another example they described was bending below the older person’s eye level, so that the person could look down instead of being addressed from above, as a way of illustrating to staff how the focus should be on the older person. Sometimes it was difficult for the personnel to know how to support the older person’s needs, which is why the first-line manager, for example, allowed staff to eat with the older people who needed help during meals. Occasionally, first-line managers also ate lunch with the older people, as a way of being a role model and focusing on the older people.

#### Encouraging attentiveness in the encounter with the older person within the frame of shared values

When leading person-centred care, the first-line managers described how they strove to encourage attentiveness in staff encounters with the older persons within the frame of shared values. They said that sharing the same vision and having the same core values were important, implying that all people were of equal value and that all people and situations were unique and must therefore be treated as such, as person-centred care sometimes requires flexibility without becoming unstructured. They said that one goal was to let the older person decide how their day should unfold, and their care should be performed. To achieve this, working with target images was described as a way to promote common values and goals, so that the older people could have good everyday lives. This could be communicated through ongoing dialogue, structured supervision at workplace meetings, and daily conversations with personnel, as ways of encouraging attentiveness when interacting with the older persons. The conversations about shared values and the central task, i.e., meeting the changing needs of the older people, were considered important to help the personnel stay focused and be constantly reminded to work in a person-centred manner with the older persons within the frame of shared values:

Yes, but every alarm is a new alarm, and I said you might have been on the toilet half an hour ago and then you got a stomach cramp and had to go to the toilet again – Shouldn’t I be allowed to go then?… What right do you have to judge how often one should go to the toilet? (Robin, FG 4)

#### Supporting older persons’ needs through resource and personnel allocation

Leading person-centred care involved allocating resources to support older persons’ needs. The first-line managers described how they, to address the changing needs of the older persons, tried to shape the resource distribution and personnel schedules. For example, a planning tool could be used to show when the older person was most in need of support. If an older person wanted to shower in the evening instead of the morning, they tried to redistribute personnel to meet the associated need. For example, if the older person only wanted to be helped to shower by the staff member, he/she felt most comfortable with, they described how they could redistribute resources so that this would be possible:

Using a particular staff member for personal interactions is important for the resident, not who is cleaning the floor, unless the resident is helping out. (Lee, I 1)

Also, tasks such as cleaning or cooking could be removed from a staff member in favour of enabling that person to be close to or work with a specific older person. In case of personnel prioritising their own needs, the first-line managers described how they could adjust the care routines or schedules in favour of the older person.

#### Promoting Understanding of the older person’s perspective through reflection

To promote an understanding of the older person’s perspective and better understand how the actions of personnel could affect older person, the first-line managers described reflecting jointly with the personnel. For example, they might ask questions instead of providing ready-made solutions, allowing the personnel to find the answers, and enhancing an understanding from the perspective of the older person. Also, spontaneous reflections were described concerning potential consequences of, for example, the older person not being allowed to continue exercising with a walker:

And then you must step back, just like Nino said before, and then you have to think with the staff, yes, but if your mother lived here, if your child went to day-care, how would you feel then? (Billie, FG 1)

Reflections concerning whether specific staff members understood an older person’s diagnosis and had ideas about how to help that person could help in designing person-centred care. The first-line managers described organising forums with external or internal professionals, for example, once a week or month, to enable group reflection on particular cases or dilemmas encountered in caring for the older people. This could promote an understanding of what person-centred care means based on the perspective of the older person.

## Discussion

The present findings revealed that person-centred leadership consists of two parts that together form a whole: *being person-centred as a leader by focusing on the relationship with the personnel* and, simultaneously, *leading person-centred care by highlighting the older persons’ needs.*

Person-centred leadership in residential care emanates from the leader as a person and presupposes a relationship with the personnel, indicating that first-line managers considered it important to be person-centred themselves. This was identified in the findings as *being person-centred as a leader by focusing on the relationship with the personnel*, where the relationships with personnel were created by, for example, being genuinely interested in the personnel and showing consideration for them as people. Being person-centred as a leader has not been explicitly addressed in previous literature on residential care, despite the prevailing consensus regarding the importance of leadership for person-centred practices [18]. However, knowing the personnel, being able to recognise the support they need, and acknowledging them as people have been reported as important for creating genuine relationships, as these may foster wellbeing among personnel, enabling them to provide good care for the older people [39–42]. This recalls person-centred theory, and McCormack and McCance [12] described how health professionals need to experience person-centredness and see themselves as equal to the people cared for. However, previous research has shown that when their personhood was not considered, personnel felt left out, abandoned, and unimportant [43]. This suggests that knowing the personnel and acknowledging their personhood are essential for creating genuine relationships. Being person-centred as a leader by acknowledging staff members’ personhood in words and deeds has the potential to promote person-centred practices, focusing on relationships by being genuinely interested, being humble, creating well-functioning teams, having confidence, being available, and including staff in decisions, as shown here. It is well known that first-line managers in this context face challenges, such as managing a large number of employees [1, 2, 27], which makes it difficult to build close relationships and be available for the personnel when needed. Other barriers have been found to influence RCF first-line managers’ ability to lead and promote person-centred care barriers such as low education, unsustainable staffing arrangements, and fundamental organisational principles leading to barriers at the person, team, and organisational levels [44]. This suggests that prerequisites and latitude for actions need to exist for first-line managers to promote person-centredness throughout the organisation, especially in times of structural challenges and strained organisations.

Person-centred leadership involves supporting staff to place the older person first while simultaneously balancing the needs of personnel. This was identified as *leading person-centred care by highlighting the older persons’ needs* when striving to enhance staff members’ understanding of the person-centred approach, as person-centredness must come from within the personnel by prioritising and focusing on the older person’s needs and preferences. The first-line managers described striving to act as a role model, enabling reflection to increase self-awareness among personnel, and encouraging attentiveness within the frame of shared values as examples of leading person-centred care with the older person in focus. McCormack and McCance [11] described that there needs to be a system for joint decision-making that includes specific values explicitly shared by everyone in the workplace. Being able to demonstrate clarity concerning beliefs and values was described as a prerequisite for person-centred care [11], supporting the present findings. Focusing on the older person while simultaneously balancing the needs of personnel was illustrated by how first-line managers directed the allocation of resources and personnel. Removing certain tasks from certain staff members and changing their schedules when the older people’s needs required it were ways of leading person-centred care by focusing on the older person. This is in line with previous findings regarding person-centred care, indicating that it is important that personnel have sufficient time to spend with the older people, allowing personnel to prioritise their needs [45]. However, in home care for older people, conflicts have been identified between person-centred work and the task-oriented system. Personnel experienced the first-line managers as leaving it to the individual staff members to resolve these conflicting demands, rather than considering them at an organisational or team level. For example, the personnel experienced a “balancing act” between the imperatives to keep the older people safe and to allow them to refuse care [43]. This indicates that the first-line manager must make necessary adjustments so that personnel can work with and for the older people, as became evident in this study. Person-centred leadership therefore entails helping personnel meet the changing needs of older people – which must drive operations – while simultaneously trying to meet the needs of personnel.

The person-centred leadership described here has various characteristics overlapping with those of other existing leadership styles. For example, features of authentic leadership [46], including being transparent and encouraging openness in sharing information, could be seen in terms of first-line managers striving to encounter staff at a personal level and being humble with them. Also, certain features could be seen in relation to ethical leadership [47], such as focusing on the relationship between the leader and the one being led, as well as being a role model to enhance mutual development. Person-centred leadership can also be related to situational leadership [48] whose fundamental principles suggest that the most successful leaders are those who adapt their leadership style to the performance readiness of the individual or group, which relates to the finding that the first-line managers strove to create functional teams, taking account of the skills and interests of personnel as well as the older person’s needs. In addition, transformational leadership [49] suggests that leaders should broaden and elevate the interests of personnel and generate awareness and acceptance of the purposes and mission of the group. Seen in relation to the present findings, transformational leadership traits are reflected in taking account of individual staff members’ unique abilities, supporting professional development, and being genuinely interested in and showing consideration for the personnel as a person. Furthermore, caritative leadership [17] highlights the relationship between the leader and the health-care professional and is motivated by the same interest: ministering to the care recipient. This relates to the findings in terms of first-line managers trying to promote person-centred care by focusing on the older person. Finally, person-centred leadership can also be seen in relation to servant leadership [50], with traits such as nurturing the personal, professional, and spiritual growth of others. This recalls the findings of our study in terms of having confidence in the unique abilities of personnel and supporting their professional development. This suggests that, although person-centred leadership has features of other leadership styles, it is novel and unique due to its specific purpose and context, i.e., promoting person-centred care. Altogether, person-centred leadership entails being and doing person-centredness as it involves both task and relational aspects, constituting a two-faceted entity that can be situated within person-centred theory [12]. In other words, person-centred leadership involves acknowledging the personhood of personnel and older people through words and actions by, for example, focusing on relationships and supporting the provision of person-centred care.

### Limitations

In this study there were only two to four participants in each focus group due to pandemic adjustments, and this small number of participants may be a potential weakness affecting the study’s credibility. The recommended sample for focus groups is five to eight participants [34]. However, for online focus groups such as those used here, it has been concluded that two to five participants are enough to obtain sufficient volume and richness of data [51]. Due to pandemic adjustments, data were gathered from both focus-group and individual interviews, which may have affected the findings. On the one hand, the aim of focus-group discussions is to share experiences and gain new insights jointly with others, which is not the aim of individual interviews. On the other hand, the data were enriched by the fact that the individual interviews gave the participants more latitude to describe their experiences in depth. The combination of the two interview forms can therefore also be seen as a strength, not only a limitation.

Some first-line managers said that they had received training in person-centred care, and this may have influenced their ability to describe person-centred care and person-centred leadership in the interviews. As in any qualitative research it is impossible to know whether the data reflect the real or ideal world. However, all participants were able to cite concrete examples of person-centred leadership, and the analyses were conducted close to the text, which strengthens the trustworthiness of our study. Still, these findings do not claim to be the only possible interpretation but can be seen as a contribution towards elaborating on the conceptualisation of person-centred leadership. This study was interested in first-line managers’ perspectives in order to further conceptualise person-centred leadership, but other perspectives are also needed, such as the registered nurses’ and other staff members’ perspectives on person-centred leadership. This is an important matter for future research to explore, which is why we recommend both interviews with personnel and observational studies of person-centred leadership. Another limitation is the risk of a gender imbalance in the results. All of the researchers were women, as were most of the participating first-line managers. There is thus a risk that gender-diverse perspectives may not have been fully explored. The sample, however, reflects the gender distribution of first-line managers in the care of older people in Sweden [27]. There is a general need for more research on gender imbalance among managers and other personnel in the care of older people.

### Clinical implications

The present findings can be used to develop clinical leadership within educational programmes, design active interventions, and further develop theoretical frameworks for person-centred leadership. The findings can also be used as a basis for reflection on the first-line managers’ leadership with a view towards building person-centred organisations.

## Conclusion

This study contributes empirical knowledge of person-centred leadership in RCFs, i.e., being and doing person-centredness as a leader, presenting concrete examples of leadership actions that first-line managers can adopt to improve person-centredness. Person-centred leadership involves both task and relational aspects, giving it two aspects that together constitute a whole. Being person-centred as a leader involves acknowledging the personhood of personnel in words and actions, giving such leadership the potential to promote person-centred practices. Person-centred leadership entails promoting personnel to meet older people’s changing needs, which must drive operations, while simultaneously trying to meet the needs of personnel. This study adds essential knowledge to an existing field in which the role of first-line managers and their leadership has been sparsely explored in terms of the concept of person-centred leadership.

## Electronic supplementary material

Below is the link to the electronic supplementary material.

Supplementary Material 1

## Data Availability

The datasets generated and/or analysed during the current study are not publicly available but are available from the corresponding author on reasonable request.
